# Gender and race equity: for a more plural science

**DOI:** 10.1590/0102-311XEN163323

**Published:** 2024-09-09

**Authors:** Vera Lucia Marques da Silva

**Affiliations:** 1 Escola Nacional de Saúde Pública Sergio Arouca, Fundação Oswaldo Cruz, Rio de Janeiro, Brasil.

## Presentation

This essay addresses gender and race equity in the sciences and their potential for the future of the scientific fields and society in general. The period from 2003 to 2010 was promising for public policies aimed at promoting equity. During this period the Department of Policies for Women and the Special Departments for Policies to Promote Racial Equality were established in Brazil ^1^. In 2005, the Women and Science Program was launched to foster studies on gender, women, and feminist studies and foment women’s participation in Sciences. In 2008, the Brazilian National Research Council (CNPq, acronym in Portuguese) included a gender equity agenda in its policies ^2^.

In 2015, the United Nations (UN) established the International Day of Women and Girls in Science to strengthen the presence and role of women in the sciences and encourage them to participate in Science, Technology, Engineering, and Mathematics (STEM) programs. Since 2010, about half of Brazil’s researchers have been women. Women accounted for 45.1% of all scientists in Latin America and the Caribbean in 2018 ^3^. Despite a certain numerical balance, sexism permeates academic life, combined with structural racism, to the point that less than 3% of professors in Brazilian graduate programs are black women with a doctoral degree ^4^.

To reflect on the effects gender and race equity can generate on the scientific and social fields, one must question the foundations of modern science and of those who produce science. Old tensions caused by imposing the Natural Sciences paradigm as “the” scientific paradigm to the detriment of the Social Sciences and Humanities - understood as lesser sciences ^5^ - persist. Notably, such tensions have been contested by new knowledge that has entered the academy and claimed its space.

Feminist epistemologies have proposed objectivity as well as other principles since the 1970s, stressing that knowledge is always situated. Given the Eurocentric character, based on the understanding of the cisheteronormative white man as a reference of humanity, which constitutes “modern” science ^6^, there is an urgent need for the participation and valuation of new research agents who, in their places of speech ^7^, produce knowledge whose constitution is made impossible by the logic of founding paradigm. How can we know the knowledge produced by transsexual women, for example, if the paradigm does not recognize their existence as worthy of being lived? In the next topic, I delve into some aspects of the feminist epistemological critique based on perspectivist feminism.

## Viewing angles

Perspectivist feminism ^8^ is a theoretical approach that began in the 1970s. Its exponents include Collins ^9^, Haraway ^10^ and Harding ^8^. Despite numerous clashes, this approach questions notions of objectivity, rationality, and universality of science. Influenced by Marxism, it understands that the material bases of human existence shape one’s vision of social reality ^7^ ^,^ ^8^ ^,^ ^9^. Thus, the supposed scientific neutrality cannot exist as any research - since its early definition - is influenced by the position its proponent occupies in the social structure. In other words, knowledge is always situated. Faced with a partial and subjective field of view, one is suspicious of the power relations that constitute the very way of doing science, which indicates that it is not only a matter of including women in the scientific field.

Therefore, a position is adopted that opposes the universality of science considered reductionist, after all, it imposes a single, phallocentric, white, and Eurocentric language as a parameter for translations, in order to obtain a single equation that accounts for the global ^10^. In this process, knowledge has been hierarchized or destroyed by the modern colonial system to impose the one which holds validity as true knowledge. Feminist epistemology seeks to destabilize hegemonic discourses by critiquing the “epistemic privilege” constructed by the colonizer via the genocide/epistemicide of the colonized ^11^: as means of politicizing these theories rather than to compete over which theory is closer to the truth, denouncing uneven power relations.

Universality is constituted by the gaze of the colonizer, who, although supposing being nowhere, also claims to be everywhere, marking “other” bodies as “different”. Groups embodied by race, gender, and sexuality were subordinated and disqualified from relevant discussions ^10^. Bodily aspects and desires were assigned identities linked to moralities related to indolence, hypersexuality, among others, in opposition to the moral ideal of the colonizer. Based on the symbolic dualisms of the colonizing system, by “embodying” others rather than himself, the colonizer reiterates nature as the place of others in opposition to his place, the subject of reason. While inscribing in nature that which must be tamed since it is the locus of the savage, culture, knowledge, and civilization are associated with reason. Thus, the other has been placed in the social hierarchy far below those who hold power, which makes them unviable as subjects of knowledge.

As an unmarked category, the “man (or god?) of science” defends seeing without being seen, representing without being represented: a fallacy of scientific objectivity since this is already in itself a position marked by the exercise of power by those who hold “the” truth. In contrast to the disembodied objectivity that generates biases, feminism proposes an embodied objectivity related to localized knowledge that can be held accountable because it is socially situated. By proposing a production of knowledge that emphasizes the leading role of black women, Xavier ^12^ exemplifies this argument by pointing out the strategic and creative view of the domestic worker who lets a cake burns so she has something to feed her child or the one who saves money to lend it at interest to her “mistress”.

However, this perspective avoids defending an essentializing view of female research subjects and their work agendas in the sense that, for example, only transsexual women can study the inequalities caused by cisgenderity. Rather, this perspective intends to recognize that the confluence of oppressions/privileges yields different perspectives on social issues, demanding plurality and dialogue to develop transformative knowledge.

## Inequalities in scientific institutions

Possible gains in gender and race equity may be envisaged. Facing the arising of complex global challenges, including social justice and democratic consolidation, involves including more women into the sciences and formulating new research questions and worldviews. Thus, reassessing the underlying power relations that hierarchizes gender and race in research institutions, universities, and funding agencies is necessary and requires the engagement of all people.

The segregation of women in the sciences is marked by the sexual division of labor - an optimal gendered and hierarchical distribution of human activities that is typical of capitalism: production (professional sphere) activities were assigned to men and reproduction (domestic sphere) activities were assigned to women ^13^. This division translates into two dimensions in scientific institutions ^14^. A horizontal one is legitimized by stereotypes that associate women with care. Thus, sciences associated with care are mostly composed of women, such as education and health; whereas men dominate the exact and technological sciences. Data indicate that, for example, 96% of professors of Naval Engineering graduate programs are men; totaling 85% in Physics programs ^15^. By being socially understood as masculine sciences, these are no longer offered as an alternative for the future of girls, thus reducing their field of possibilities for professional choice and development. Unsurprisingly, only 30% of female higher education students worldwide entered STEM programs, representing only 35% of all students enrolled in these careers ^16^.

Studies show the obstacles of female scientists in reaching positions high-rank and high-prestige throughout their careers. The pinnacle of their careers remains masculine (even for “feminine” sciences) thanks to the “scissors effect”: the gradual decrease in figures of women as careers progress ^17^. This vertical segregation is alien to the social reality of women, on whom the responsibility for reproductive work falls greatly and to whom earnings lower than their colleagues are reserved, negatively influencing their access to job opportunities.

A survey carried out from 2017 to 2018 indicates that the reduction in women’s productivity occurs from the birth of their offspring to at least the child’s fourth year. This scenario can be observed in many fields of knowledge worldwide ^18^. Regarding average real monthly income, the Brazilian women employed in the third quarter of 2022, for example, earned 21% less than men ^19^.

Although women are majority among professionals of Public Health, their difficulties are noteworthy ^20^. A study on the distribution of CNPq funding projects from 2004 to 2006, for example, found that 43 women received a grade 2 scholarship (initial category), against 32 men. Moreover, four women and 16 men received grade 1A scholarships (the highest category), unveiling a marked inequality ^21^.

Thus, girls and women face significant losses, but so does society. A scientific culture marked by sexism, racism and transphobia hinders global development. Economic gains can be indicated as the positive relation between financial resilience and a greater presence of women in institutions and the formulation of financial policies and improved performance and profitability across financial digital innovation companies and the corporate sector in which female leaders predominate ^22^.

## Final considerations

To think about gender and race equity in the sciences refers to thinking of new research approaches and agendas as well as of inclusive teaching and research institutions in which the diversity of women (who endure multiple systems of oppressions) can have a place, be acknowledged, and produce knowledge that is only possible to those who experience the effects of the intersection between these oppressions, hence the centrality of adopting an intersectional perspective to understand how these crossings produce and reproduce inequalities ^23^. Far from romanticizing the place of the oppressed, reality must be approached in its complexity from different points of view as each offers an inevitably partial perspective ^7^. Added to this is the necessary restitution of denied humanities ^12^, such as that of black, Indigenous, transgender, and non-binary people, by including and acknowledging their role as agents of knowledge, which would involve producing statistical data. The lack of statistics indicating the number of transgender scientists, for example, indicates this impossibility of their existence in cisgender eyes, which demands restoration.

It also distances itself from essentialisms: what is valued is the multiplicity of situated knowledge placed in critical dialogue toward justice and social development. After all, the defense of feminist policies dispenses with the condition of identifying as a woman ^24^. As a political position, adhesion involves choice and action. Therefore, I invite all people to contribute to promoting a scientific ethos that values diversity and the constant, dare I say, renunciation of privileges, whether they come from hierarchies of gender, race, or social class, in favor of a more just and democratic society.
