# Supplementary material for: Gender and race equity: for a more plural science
Source: Cad Saude Publica. 2024 Sep 9;40(8):e00163323. doi: 10.1590/0102-311XEN163323 (PMC11386528; doi:10.1590/0102-311XEN163323)
Supplement: Translation

# Translation: Equidade de gênero e raça: por uma ciência mais plural

**DOI:** 10.1590/0102-311XPT163323

**Authors:** Vera Lucia Marques da Silva

**Affiliations:** 1 Escola Nacional de Saúde Pública Sergio Arouca, Fundação Oswaldo Cruz, Rio de Janeiro, Brasil.

## Apresentação

Este ensaio aborda o tema da equidade de gênero e raça nas ciências e suas potencialidades para o futuro do campo científico e da sociedade em geral. O período de 2003 a 2010 foi promissor para políticas públicas a esse respeito. No Brasil, constituíram-se Secretarias de Políticas para Mulheres e de Promoção da Igualdade Racial ^1^. Em 2005, foi lançado o Programa Mulher e Ciência para fomentar estudos sobre gênero, mulheres e feminismos e promover a participação das mulheres nas ciências. Em 2008, o Conselho Nacional de Desenvolvimento Científico e Tecnológico (CNPq) inseriu a agenda da equidade de gênero em suas políticas ^2^.

Em 2015, a Organização das Nações Unidas (ONU) instituiu o Dia Internacional de Mulheres e Meninas na Ciência para fortalecer a presença e atuação das mulheres nas ciências, além de incentivá-las a se inserir em Ciências, Tecnologia, Engenharia e Matemática (STEM, acrônimo em inglês). Em números, desde 2010, cerca de metade dos pesquisadores brasileiros são mulheres. Na América Latina e Caribe, representavam 45,1% em 2018 ^3^. Apesar de certo equilíbrio numérico, o sexismo atravessa a vida acadêmica, combinado ao racismo estrutural, de modo que menos de 3% dos docentes da pós-graduação brasileira são doutoras negras ^4^.

Refletir sobre os efeitos que a equidade de gênero e raça podem gerar nos campos científico e social leva a questionar os alicerces da ciência moderna, assim como quem produz ciência. Velhas tensões, provocadas pela imposição do paradigma das Ciências Naturais como “o” paradigma científico, em detrimento das Ciências Humanas e Sociais, vistas como menores ^5^, permanecem em curso, problematizadas por novos saberes que adentram a academia e pleiteiam espaço.

Epistemologias feministas tencionam princípios como o da objetividade desde a década de 1970, apontando que o conhecimento é sempre situado. Diante do caráter eurocentrado, baseado no entendimento do homem branco cisheteronormativo como referência de humanidade, que constitui “a” ciência moderna ^6^, urge a entrada e o reconhecimento de novos(as) agentes de pesquisa que, de seus lugares de fala ^7^, produzam conhecimentos cuja constituição é impossibilitada pela lógica do paradigma fundante. Como conhecer os saberes produzidos por mulheres transexuais, por exemplo, se o paradigma não reconhece sua existência como digna de ser vivida? No próximo tópico, aprofundo alguns aspectos da crítica epistemológica feminista, a partir do feminismo perspectivista.

## Ângulos de visão

O feminismo perspectivista ^8^ é uma abordagem teórica que teve início nos anos de 1970. Entre suas expoentes, pode-se citar Collins ^9^, Haraway ^10^ e Harding ^8^. A despeito de inúmeros embates, questionam-se as noções de objetividade, racionalidade e universalidade da ciência. Influenciado pelo marxismo, entende que as bases materiais de existência humana conformam a visão que se tem da realidade social ^7^ ^,^ ^8^ ^,^ ^9^. Isso impossibilita a neutralidade científica, já que qualquer pesquisa, desde sua definição inicial, é influenciada pela posição ocupada por seu/sua proponente na estrutura social. Ou seja, trata-se de um conhecimento sempre situado. Diante de um campo de visão parcial e subjetivo, suspeita-se das relações de poder que constituem o próprio modo de fazer ciência, o que indica não se tratar apenas da inclusão das mulheres no campo científico.

Com isso, adota-se uma posição antagônica à universalidade própria da ciência, considerada reducionista, pois impõe uma única linguagem (falocêntrica, branca e eurocentrada) como parâmetro para produção de traduções, visando obter uma única equação que dê conta do global ^10^. Nesse processo, saberes foram hierarquizados ou destruídos pelo sistema colonial moderno para impor aquele que tem validade como conhecimento verdadeiro. A epistemologia feminista busca desestabilizar discursos hegemônicos pela crítica ao “privilégio epistêmico” construído pelo colonizador com o genocídio/epistemicídio dos/as colonizados/as ^11^, não para discutir sobre qual teoria está mais próxima da verdade, mas para politizá-las, denunciando relações desiguais de poder.

A universalidade se constitui por meio do olhar do colonizador, que, ao supor não estar em lugar nenhum, alega estar igualmente em toda parte, marcando os “outros” corpos como “diferentes”. Grupos corporificados por raça, gênero e sexualidade foram subalternizados e desqualificados para discussões relevantes ^10^. A aspectos e desejos corporais atribuiu-se identidades, atreladas a valores morais relacionados à indolência, hipersexualidade, entre outras, opostas ao ideal moral do colonizador. A partir de dualismos simbólicos do sistema colonial, ao “dar corpo” aos outros e não a si, o colonizador reitera a natureza como o lugar dos outros em oposição ao seu lugar, de sujeito da razão. Enquanto na natureza se inscreve aquilo que precisa ser domado, haja vista que é o lócus do selvagem, à razão se associam cultura, conhecimento, civilização. Assim, os outros foram posicionados na hierarquia social como aquém daqueles que detêm poder, o que os inviabiliza como sujeitos do conhecimento.

Como categoria não marcada, o “homem (ou deus?) da ciência” pretende enxergar sem ser visto, representar sem ser representado: uma falácia da objetividade científica, uma vez que esse já é, em si, um posicionamento, marcado pelo exercício do poder de quem detém “a” verdade. Contrapondo-se à objetividade descorporificada, que gera vieses, a objetividade proposta pelo feminismo é corporificada: se relaciona a saberes localizados, passíveis de serem responsabilizados, porque socialmente situados. Ao propor uma produção de conhecimento que enfatize o protagonismo das mulheres negras, Xavier ^12^ exemplifica essa argumentação apontando a visão estratégica e criativa da empregada doméstica que deixa um bolo queimar para ter o que dar de comer ao filho, ou da que economiza dinheiro para emprestá-lo a juros a sua “patroa”.

Não se trata, todavia, da defesa de uma visão essencializadora de sujeitas de pesquisa e suas agendas de trabalho, no sentido de que, por exemplo, apenas mulheres transexuais poderiam estudar as desigualdades provocadas pela cisgeneridade. O que se pretende é o reconhecimento de que a confluência de diferentes opressões/privilégios produz perspectivas distintas sobre questões sociais. A demanda é por pluralidade e diálogo para o desenvolvimento de conhecimentos transformadores.

## Desigualdade em instituições científicas

Possíveis ganhos da equidade de gênero e raça podem ser entrevistos. O enfrentamento dos complexos desafios globais que se apresentam, como justiça social e consolidação democrática, passa pela incorporação de mais mulheres nas ciências, com a formulação de novas questões de pesquisa e visões de mundo. Logo, rever relações de poder que fundamentam hierarquias de gênero e raça nas instituições de pesquisa, universidades e agências de fomento é mister e requer engajamento de todos/as.

A segregação das mulheres nas ciências é marcada pela divisão sexual do trabalho - distribuição ideal generificada e hierarquizada das atividades humanas própria do capitalismo: atividades de produção (esfera profissional) foram atribuídas aos homens e as de reprodução (esfera doméstica) destinadas às mulheres ^13^. Tal divisão se traduz em instituições científicas em duas dimensões ^14^. Uma, horizontal, legitimada por estereótipos que associam mulheres ao cuidado. Consequentemente, ciências associadas ao cuidado, como educação e saúde, são majoritariamente constituídas por mulheres, enquanto nas exatas e tecnológicas predominam homens. Dados indicam que na Engenharia Naval, por exemplo, 96% dos docentes de pós-graduação são homens, e, na Física, 85% ^15^. Esses espaços, ao serem socialmente lidos como masculinos, deixam de ser oferecidos como alternativa de futuro para meninas. Reduz-se, portanto, seu campo de possibilidades de escolha e desenvolvimento profissional. Não à toa, entre as discentes de ensino superior no mundo, somente 30% ingressaram em STEM, o que representa apenas 35% do total de matriculados nessas carreiras ^16^.

Estudos demonstram a dificuldade das cientistas em alcançar postos de poder e prestígio ao longo de suas carreiras. O ápice da carreira ainda é masculino, mesmo em ciências tidas como femininas, graças ao “efeito tesoura”: a redução gradativa da presença dessas, à medida que a carreira progride ^17^. Essa segregação - vertical - é alheia à realidade social das mulheres sobre as quais a responsabilidade pelo trabalho reprodutivo recai sobremaneira, além de obterem proventos menores que seus colegas, o que influencia negativamente no acesso a oportunidades de trabalho.

Levantamento realizado entre 2017 e 2018 sinaliza que a redução da produtividade das mulheres ocorre do nascimento de seu/sua filho/a até, no mínimo, seus quatro anos. Esse cenário é identificado em diferentes áreas do conhecimento mundo afora ^18^. Quanto ao rendimento médio real mensal, as brasileiras “ocupadas” no 3º trimestre de 2022, por exemplo, ganharam 21% menos que os homens ^19^.

Apesar de o campo da Saúde Coletiva ser majoritariamente constituído por mulheres, suas dificuldades são uma realidade ^20^. Estudo sobre a distribuição de bolsas CNPq entre 2004 e 2006, por exemplo, identificou que 43 mulheres receberam bolsa 2 (categoria inicial), ante 32 homens. Já na categoria 1A (o nível mais alto), quatro mulheres e 16 homens foram contemplados, denunciando uma acentuada desigualdade ^21^.

Evidente, portanto, que meninas e mulheres têm perdas significativas, mas a sociedade também. A cultura científica marcada por sexismo, racismo e transfobia dificulta o desenvolvimento global. Ganhos econômicos podem ser indicados, como a relação positiva entre resiliência financeira e maior presença de mulheres em instituições e na formulação de políticas financeiras; e melhor desempenho e rentabilidade entre empresas de inovação digital financeira e do setor corporativo no qual predominam mulheres líderes ^22^.

## Considerações finais

Pensar em equidade de gênero e raça nas ciências é pensar tanto em novas abordagens e agendas de pesquisa quanto em instituições de ensino e pesquisa inclusivas, em que diversas mulheres, atravessadas por múltiplos sistemas de opressões, possam ter lugar, reconhecimento e gerar conhecimentos que apenas quem vivencia os efeitos da interseção entre essas opressões é capaz de produzir. Daí a centralidade da adoção de uma ótica interseccional para compreender como esses atravessamentos produzem e reproduzem desigualdades ^23^. Longe de romantizar o lugar do oprimido, admite-se que a realidade - em sua complexidade - precisa ser depreendida de diferentes ângulos, pois de cada qual tem-se uma perspectiva que é sempre parcial ^7^. Soma-se a necessária restituição de humanidades negadas ^12^, como a de pessoas negras, indígenas, transgêneras e não-binárias, a partir de sua inclusão e reconhecimento de sua atuação como agentes de conhecimento, o que passa pela produção de dados estatísticos. A ausência de estatísticas sobre o número de cientistas transgêneras, por exemplo, é indicativa dessa impossibilidade de existência aos olhos cisgêneros, que precisa ser estabelecida.

Distancia-se também de essencialismos: se preza pela multiplicidade de conhecimentos situados, postos em diálogo crítico, para concretização de justiça e desenvolvimento social. Afinal, para a defesa de políticas feministas prescinde-se de se identificar como mulher ^24^. Como posição política, a adesão envolve escolha e ação. Logo, convido todos/as/es a contribuírem na promoção de um *ethos* científico que valorize a diversidade e a constante, ouso dizer, renúncia de privilégios, sejam eles oriundos de hierarquias de gênero, raça ou classe social, em prol de uma sociedade mais justa e democrática.
